# Undergoing Orthopaedic Day Surgery: What Factors Are Associated With patients' Feeling of Safety and Their Recovery?

**DOI:** 10.1111/jocn.17552

**Published:** 2024-11-11

**Authors:** Fanny Larsson, Åsa Engström, Ulrica Strömbäck, Silje Rysst

**Affiliations:** ^1^ Department of Health, Education and Technology, Division of Nursing and Medical Technology Luleå University of Technology Luleå Sweden

**Keywords:** day surgery, feeling safe, nursing, postoperative recovery

## Abstract

**Aim:**

The study aimed to examine factors associated with the perceived feeling of safety and postoperative recovery in patients who have undergone orthopaedic day surgery under regional anaesthesia.

**Design:**

The design was quantitative, descriptive, and cross‐sectional. The study participants comprised a consecutive sample (*n* = 209) of patients who underwent orthopaedic day surgery under regional anaesthesia.

**Methods:**

A questionnaire was sent to the home addresses of the study population approximately 3 weeks postoperatively. The questionnaire included the Feeling Safe During Surgery Scale (FSS), the Swedish version of the post‐discharge surgical recovery scale (S‐PSR), and questions concerning background variables. Multivariate regression models were used to examine the association of different variables with both feeling safe and postoperative recovery.

**Results:**

The only factor associated with the feeling of safety was preoperative anxiety; higher levels of preoperative anxiety were associated with lower levels of perceived safety during surgery. The factors associated with postoperative recovery were the recovery process itself and the patient's feeling of safety. Higher levels of postoperative anxiety were associated with a lower level of postoperative recovery. Higher levels of perceived safety during surgery were associated with higher postoperative recovery.

**Conclusion:**

The perceived feeling of safety in the perioperative period could not be explained by factors such as age, gender, or level of education. Based on the results of this study, postoperative recovery was associated with the perceived feeling of safety in the perioperative period. Anxiety in the perioperative period was associated with patients' perceived feeling of safety and their postoperative recovery. Thus, this study's results emphasise the importance of ensuring that people undergoing surgery feel safe to promote their recovery. Based on previous research, the nurse–patient relationship seems to be an important part of making patients feel safe, which ultimately affects their recovery.

**Implications for the Profession/and or Patient Care:**

This study examines the association between perceived feeling of safety in the perioperative period and patients' postoperative recovery after undergoing orthopaedic day surgery under regional anaesthesia. Previous research has shown that the nurse–patient relationship and patients' possibilities to participate in their care are important for them to feel safe. This study further emphasises the importance of fostering relationships in the perioperative period and making patients an active part in decision‐making, as it may positively impact their recovery. Creating a feeling of safety for the patient should be prioritised, as it benefits their perioperative experience and postoperative recovery.

**Reporting Method:**

This research is reported in accordance with the STROBE guidelines.

**Patient or Public Contribution:**

No patient or public contribution.

**Summary**

- The findings from this study provide knowledge on some factors associated with patients' perceived feeling of safety and postoperative recovery when undergoing orthopaedic day surgery.
- The findings show that a higher perceived feeling of safety in the perioperative period is associated with a higher level of postoperative recovery after orthopaedic day surgery.

## 1 Introduction

Over the past decade, research has increasingly focused on patients' perceptions of safety within healthcare. In surgical care, feeling safe is characterised by attributes such as participation, control, and presence. People undergoing orthopaedic day surgery are particularly vulnerable; their recovery can be prolonged, and much of it occurs independently, without the support of healthcare professionals.

### 1.1 Background

Day surgery, a procedure in which a patient is admitted, undergoes surgery and is discharged the same day, has become more common over the last decades (Darwin 2016; Organisation for Economic Co‐Operation and Development [OECD] 2023). The benefits associated with day surgery, including early mobilisation, lower risk for nosocomial infections as well as cost‐effectiveness, are often highlighted when describing day surgery (Darwin 2016; Odom‐Forren et al. 2015; OECD 2023). Additionally, the fact that patients often prefer day surgery is highlighted as beneficial (Halding et al. 2021; Thoen et al. 2024). Although day surgery has its advantages, it can be challenging for patients, as the efficiency of the procedure and process may leave them feeling like objects, and access to healthcare post‐discharge may be limited (Berg, Årestedt, and Kjellgren 2013; Odom‐Forren et al. 2015; Odom‐Forren, Reed, and Rush 2017).

The most common surgical procedures in Sweden are orthopaedic with surgeries on feet, knees, shoulders, and hands accounting for a major part (Socialstyrelsen 2023). Patients undergoing orthopaedic surgery have a higher likelihood of experiencing pain postoperatively compared to patients undergoing other surgical procedures (Odom‐Forren et al. 2015). Further, the postoperative recovery of patients undergoing orthopaedic inpatient surgery was reported to be worse than that of people undergoing other types of surgeries. Unlike patients undergoing other surgical procedures, orthopaedic patients reported a decline in psychological functioning 1 month postoperatively (Forsberg et al. 2018). Regional anaesthesia is common for orthopaedic procedures, particularly in European countries (Cozowicz, Poeran, and Memtsoudis 2015). Benefits of regional anaesthesia may include a reduction of several discomforting symptoms such as postoperative pain, vomiting, and fatigue (Nilsson et al. 2019). However, people undergoing surgery with regional anaesthesia might have concerns about potential pain, remaining conscious during their procedure, or witnessing the surgical procedure (Stamenkovic et al. 2018). In addition, the experience of postoperative fatigue might still be present even after undergoing minor surgery under regional anaesthesia (Nilsson et al. 2019).

Postoperative recovery may be divided into three phases as follows: (1) The immediate/early phase, which lasts up to the point when a patient is awake and able to protect their vital functions; (2) The intermediate phase, which refers to the point where patients who have undergone day surgery may be discharged. At this point, patients have regained more functions and experience less dizziness and (3) The long‐term/late phase, which lasts for several days and ends when the process of recovery is completed (Bailey et al. 2019; Marshall and Chung 1999; Nilsson et al. 2020; Steward and Volgyesi 1978). Nilsson et al. (2020) have defined postoperative recovery in a day‐surgical context as ‘a dynamic process leading to an experience of a new stable state’ (Nilsson et al. 2020). The recovery process starts during the preoperative period and is influenced by different dimensions, including social, habitual, and physiological factors. The new stable state marking the end of the recovery process does not necessarily indicate a return to the preoperative condition. It may result in the person being either more or less functional than before the surgery. Moreover, an important part of reaching a new stable state involves adjusting to one's new condition (Nilsson et al. 2020). Patients' experiences of postoperative recovery may be influenced by different factors such as age, gender, mental health status, and health literacy (Jaensson, Dahlberg, and Nilsson 2019). Further, the perceived feeling of safety during the perioperative period may positively impact patients' postoperative recovery (Dahlberg et al. 2018).

The concept of feeling safe from the patient's perspective has been described in different healthcare settings (Mollon 2014; Péculo‐Carrasco et al. 2020; Pelto‐Piri et al. 2019; Silverglow et al. 2021; Wassenaar, Schouten, and Schoonhoven 2014). In a perioperative setting, the perceived feeling of safety is described as the absence of feeling worried or threatened. The concept of feeling safe during the perioperative period encompasses three attributes, namely: (1) Participation, which involves patients' desire to take an active part in decision‐making; (2) Control, which entails patients' ability to trust the competence of the staff and (3) Presence, which refers to the presence of both healthcare staff and significant others (Larsson et al. 2023).

In summary, people undergoing day surgery may encounter a challenging postoperative recovery, with limited healthcare contact after discharge. Orthopaedic procedures are Sweden's most common surgical procedures, with many performed as day surgeries. Apart from being a common procedure, patients undergoing orthopaedic procedures constitute a particularly vulnerable group, often experiencing postoperative difficulties, including postoperative pain, to a greater extent than those undergoing other surgical procedures. Various factors, such as age, gender, and the perceived feeling of safety, may influence a person's postoperative recovery. However, to the best of our knowledge, there are limited studies investigating the association between feelings of safety during the perioperative period and postoperative recovery specifically in orthopaedic day surgery patients.

## 2 The Study

### 2.1 Aim

The aim of this study was to examine factors associated with the perceived feeling of safety and postoperative recovery in patients who have undergone orthopaedic day surgery under regional anaesthesia.

## 3 Methods

### 3.1 Design

The study design was quantitative and cross‐sectional. The reporting in this study follows the STrengthening the Reporting of OBservational studies in Epidemiology (STROBE) checklist (Vandenbroucke et al. 2007).

### 3.2 Participants and Setting

The study participants consisted of a consecutive sample (*n* = 209) of patients who underwent orthopaedic day surgery under regional anaesthesia between March 2022 and January 2024. Inclusion criteria were elective orthopaedic day surgery with regional anaesthesia, 18 or older, and understanding and speaking Swedish.

Participants were recruited from two surgery wards in separate hospitals in northern Sweden. The first ward specialises in elective surgeries, primarily orthopaedic, both inpatient and outpatient. Around 20 to 30 day surgeries are performed each week, many under regional anaesthesia. The second ward, situated in a county hospital within a different region, performs orthopaedic day surgeries weekly alongside elective and emergency surgeries across various surgical disciplines. The number of orthopaedic day surgeries performed varies each week.

At the surgical wards where participants were recruited, patients scheduled for surgery met an orthopedist at an appointment before the surgery. On the day of surgery, patients meet their orthopaedic surgeon upon arrival at the ward and again before being discharged from the Post‐Anaesthesia Care Unit (PACU). During surgery, a team consisting of a nurse anaesthetist, an operating room nurse, and a licensed practical nurse takes care of the patient. An anesthesiologist is present when administering regional anaesthesia and maintains overall responsibility for the patient, although they may not be present in the operating room throughout surgery. In the PACU, patients are cared for by nurse anaesthetists and licensed practical nurses, both before and after surgery. Patients typically remain in the PACU for phases I and II of their recovery. Follow‐up appointments after discharge are rare.

### 3.3 Data Collection

Addresses of patients whose characteristics met the inclusion criteria were collected from the surgical planning document. An invitation letter and a questionnaire were sent to the home addresses of the study population, which consisted of 441 patients, approximately 3 weeks postoperatively. The invitation letter contained information regarding the aims and procedures of the study. Patients willing to participate filled out the questionnaire and sent it back; thus, a filled‐out questionnaire was seen as informed consent. A wave of reminders was sent out after 2 weeks to non‐respondents.

The questionnaire included the Feeling Safe During Surgery Scale (FSS) (Larsson et al. 2021) the Swedish version of the post‐discharge surgical recovery scale (S‐PSR) (Berg et al. 2010), and questions concerning background variables (Table 1). Participants were further asked to rate their preoperative level of anxiety and pain and their postoperative level of pain, nausea, and anxiety using the Numeric Rating Scale (NRS). Both FSS and S‐PSR are evaluated regarding validity and reliability, and are measuring the constructs under measure (Berg et al. 2010; Rysst and Larsson 2024). Additionally, the questionnaire contained open‐ended questions targeting patients' experiences regarding feelings of safety and day surgery. The result from the open‐ended questions is reported in a separate study (Larsson et al. 2024).

**TABLE 1:** Participants' characteristics.

	*N*	%
Gender (*n* = 209)		
Men	80	38.3
Women	129	61.7
Level of education (*n* = 209)		
High school	44	21.1
Upper secondary school	74	35.4
University < 3 years	37	17.7
University > 3 years	54	25.8
Occupation (*n* = 209)		
Working	92	44
Unemployed	4	1.9
Retired	113	54.1
Student	0	0
Marital status (*n* = 207)		
Single	30	14.5
Married/partner	159	76.8
Widow/widower	18	8.7
Type of surgery (*n* = 203)		
Hand	65	32
Arm	22	10.8
Foot	89	43.8
Shoulder	15	7.4
Knee	12	5.9
Previous surgical experience (*n* = 208)		
No	149	71.6
Yes	59	28.4
Use of sedatives during surgery (*n* = 206)		
Yes	96	46.6
No	71	34.5
Don't know	39	18.9

	Mean (SD)
Preoperative pain* (*n* = 207)	4.29 (2.66)
Preoperative anxiety* (*n* = 208)	2.56 (2.11)
Postoperative pain* (*n* = 200)	2.09 (1.91)
Postoperative anxiety* (*n* = 203)	1.76 (1.70)
Postoperative nausea* (*n* = 202)	1.50 (1.50)

* Using the Numeric Rating Scale (NRS) scale (1–10).

From the study population of 411 individuals, 209 response envelopes were received, resulting in a response rate of 51%. To determine sample size and avoid a type II error (Beck and Polit 2017) a power analysis using the ClinCalc.com online statistical program was performed. The power analysis was based on the results from a pilot study (Andersson et al. 2020), which used the visual analogue scale (VAS) to measure preoperative anxiety and compared means for women and men. The power analysis indicated that to obtain an 80% power with an alpha of 0.05, a total of 188 participants were required for the study.

### 3.4 Data Analysis

The data were analysed using IBM SPSS v. 29 (IBM, Armonk, NY). The significance level was set to 5%. The data were analysed descriptively to provide an overview of the participants.

To analyse the overall experience of safety, a feeling safe score (FSS score) was determined by calculating the participant's sum score of items 1–10 of the 10‐item version of the FSS (cf. Rysst and Larsson 2024). The final range score is then 10–100, with a higher score indicating a higher level of feeling safe. To analyse the overall recovery, a recovery score (PSR score) was calculated by dividing the participant's sum score of S‐PSR with the highest possible score of S‐PSR and multiplying the result by 100. The final range score is thus 10–100, with higher scores indicating positive postoperative recovery (cf. Berg et al. 2010).

Multivariate regression models using the backward elimination method were used to examine the association of different variables with feeling safe and postoperative recovery. Tolerance, the variance inflation factor (VIF), and the Durbin‐Watson test were evaluated to assess multicollinearity. The first regression model used the FSS score as a dependent variable. The initial independent variables used were gender, age, level of education, type of surgery, previous experience, and preoperative level of pain and anxiety. The second regression model used the PSR score as a dependent variable. The initial independent variables were gender, age, level of education, type of surgery, previous experience, preoperative level of pain and anxiety, and feeling safe score. Dummy variables were created for Gender (Women = 1, Male = 0), Previous surgical experience (Previous experience = 1, No previous experience = 0), and Type of Surgery (Knee‐surgery as the reference group) to indicate different groups, with one category as the reference group. This is due to the categorical nature of these variables.

### 3.5 Ethical Considerations

The study received approval from the Ethical Review Board in Sweden (Reference Numbers: 2020‐04703, 2020‐07129, and 2022‐01246‐02). Prior to the study's implementation, and collection of addresses to participants, consent was obtained from unit managers and heads of surgical wards. Participants were granted confidentiality and provided with detailed information regarding the study's aim and procedures. They were informed of their voluntary participation rights, with the option to withdraw at any time without repercussion or the need for justification, thus ensuring informed consent was obtained from all participants.

## 4 Results

### 4.1 Description of Participants

The participants' age (*n* = 209) ranged from 22 to 92 years (mean 64.1 years, SD 12.66 years). Participants' characteristics are presented in Table 1.

### 4.2 Perceived Feeling of Safety and Postoperative Recovery

Participants' perceived feeling of safety during the perioperative period was high. Means and SD of the perceived feeling of safety pre‐, peri‐, and postoperatively, as well as the FSS score and PSR score, are displayed in Table 2.

**TABLE 2:** Perioperative safety and postoperative recovery.

	*N*	Mean (SD)
Feeling of safety before surgery	208	8.62 (1.94)
Feeling of safety during surgery	204	9.25 (1.35)
Feeling of safety after surgery	208	8.81 (1.699)
Feeling of safety post‐discharge	208	8.28 (1.946)
FSS score	195	91.76 (10.63)
PSR score	190	65.69 (16.09)

### 4.3 Associations With the Perceived Feeling of Safety

The first regression model (Table 3) used the FSS score as the dependent variable. The regression model with backward elimination explained 14.2% of the variance. Previous surgical experience (*p* = 0.047) and preoperative anxiety (*p* = 0.001) were associated with the perceived feeling of safety during the perioperative period. Thus, higher levels of preoperative anxiety are associated with lower levels of perceived safety. Previous surgical experience is associated with higher levels of perceived safety. The mean tolerance was 0.9, the mean VIF was 1.11, and the Durbin Watson was close to 2, suggesting no significant multicollinearity or intercorrelation in the regression model (Hair et al. 2019).

**TABLE 3:** Regression model. Dependent variable: FSS Score.

	Unstandardized B‐coefficients (Std. Error)	Standardised Beta‐coefficient	*p*	Tolerance	VIF
Gender – Female	0.49 (1.58)	0.02	0.76	0.92	1.09
Age	0.04 (0.06)	0.05	0.502	0.85	1.18
Level of education	−1.14 (0.75)	−0.12	0.13	0.84	1.19
Previous surgical experience—Yes	3.41 (1.70)	0.14	0.047*	0.93	1.07
Preoperative anxiety	−1.20 (0.36)	−0.23	0.001*	0.94	1.06
Surgery – Hand	−3.11 (2.55)	0.09	0.065	0.90	1.12
Surgery – Arm	3.31 (2.55)	0.09	0.196	0.92	1.09
Intercept	94.74 (5.12)		< 0.001*		
*N*	187				
*R* ^2^	0.142				
Adj. *R* ^2^	0.114				
Durbin Watson	2.2				

* *p* < 0.05.

Abbreviation: FSS, Feeling Safe During Surgery Scale.

### 4.4 Associations With Postoperative Recovery

The second regression model (Table 4) used the PSR score as the dependent variable. The regression model with backward elimination explained 13.7% of the variance. FSS score (*p* = 0.035) and preoperative anxiety (*p* < 0.001) were the only variables that significantly contributed to the model. Thus, higher levels of preoperative anxiety are associated with a lower level of postoperative recovery, and higher levels of perceived safety during surgery are associated with a higher level of postoperative recovery. The mean tolerance was 0.91, the mean VIF was 1.1, and the Durbin‐Watson was close to 2, suggesting no significant multicollinearity or intercorrelation in the regression model (Hair et al. 2019).

**TABLE 4:** Regression model. Dependent variable: PSR score.

	Unstandardized B‐coefficients (Std. Error)	Standardised Beta‐coefficients	*p*	Tolerance	VIF
FSS Score	0.24 (0.11)	0.17	0.035*	0.86	1.16
Level of education	1.69 (1.09)	0.12	0.123	0.91	1.09
Previous surgical experience—Yes	3.22 (2.61)	0.11	0.219	0.95	1.05
Type of surgery—Hand	3.18 (2.58)	0.09	0.219	0.94	1.07
Preoperative anxiety	−2.10 (0.57)	0.28	< 0.001*	0.90	1.11
Intercept	42.94 (11.69)		< 0.001*		
*N*	171				
*R* ^2^	0.137				
Adj. *R* ^2^	0.111				
Durbin Watson	1.99				

* *p* < 0.05.

Abbreviations: FSS, feeling seafe during surgery scale; PSR, post‐discharge surgical recovery scale.

## 5 Discussion

This study aimed to describe factors associated with the perceived feeling of safety and postoperative recovery in patients who underwent day surgery under regional anaesthesia. The results show that higher levels of preoperative anxiety were associated with lower levels of perceived safety during surgery. Previous surgical experience was associated with higher levels of perceived feelings of safety. Further, higher levels of preoperative anxiety were associated with a lower level of postoperative recovery, while higher levels of perceived safety during surgery were associated with a higher level of postoperative recovery.

Most participants felt safe during the perioperative period. This feeling was evident in the calculated FSS score and when participants were asked to rate their perceived feeling of safety pre‐, peri, postoperatively, and after discharge. When calculating the recovery score (PSR score), the mean was 65.69 (out of 100). This indicates that for most people, the recovery process is not complete this soon after surgery. This is in line with Stessel et al. (2021) who examined postoperative recovery after day surgery 1 month postoperatively (28 days). Their results indicate that the type of surgery predicted recovery from orthopaedic procedures, with arthroscopic shoulder surgery especially associated with poorer recovery. Previous studies have described patients' experiences of postoperative recovery after orthopaedic day surgery. They have found that information provided before discharge did not meet patients' needs regarding self‐management after discharge (Halding et al. 2021) and questions about the period of recovery do not arise before they have already been discharged (Halding et al. 2021; Larsson et al. 2022). Patients have described a need for confirmation that the recovery is proceeding as expected and a wish for a follow‐up visit (Larsson et al. 2022).

The first regression model used the perceived feeling of safety (FSS score) as the dependent variable. The results show that preoperative anxiety and previous surgical experience were associated with the perceived feeling of safety during the perioperative period. The second model used the estimated recovery (PSR score) as the dependent variable. The model showed that preoperative anxiety and the perceived feeling of safety (FSS score) were the only factors that were associated with postoperative recovery. To some extent, this result contradicts previous findings. When the quality of recovery after day surgery was examined 4 days postoperatively, older age and employment status predicted poorer postoperative recovery (Stessel et al. 2015). When examining the postoperative recovery 1 month (28 days) postoperatively after painful day surgery—such as hemorrhoid surgery, inguinal hernia repair, arthroscopic knee, and arthroscopic shoulder surgery—occupation and female gender were predictors of poorer postoperative recovery (Stessel et al. 2021; Jaensson, Dahlberg, and Nilsson 2019) suggest that patients' gender, age, mental health status, and health literacy should be evaluated, as these factors may influence postoperative recovery.

Preoperative anxiety and its association with both perceived safety and postoperative recovery is somewhat consistent with previous studies, which show that anxiety in the perioperative period is common among surgical patients. It may affect their postoperative period, influencing factors such as postoperative mood, perceived pain, and an increased need for analgesics (Aust et al. 2018; Munafò and Stevenson 2001; Stamenkovic et al. 2018). Nurses play an important role in reducing patients' anxiety as they are in direct contact with patients throughout the perioperative period. In addition, nurses are trained to provide patients with emotional care and support (Agüero‐Millan, Abajas‐Bustillo, and Ortego‐Maté 2023), and nurse interventions such as educational and motivational interviews can reduce preoperative anxiety in patients (Ruiz Hernández et al. 2021).

The result of this study shows that patients' perioperative experience of feeling safe is associated with their postoperative recovery, which is in line with (Dahlberg et al. 2018). Previous research suggests that for patients to experience good perioperative care, they need to feel safe (Mako, Svanäng, and Bjerså 2016). This can be achieved through accessible and reliable care, where patients are engaged by receiving relevant information and can influence their care (Mako, Svanäng, and Bjerså 2016). Nurses play a crucial role in promoting patients' sense of safety, both during hospitalisation and in surgical settings (Groves, Bunch, and Kuehnle 2023; Larsson et al. 2022; Wassenaar, Schouten, and Schoonhoven 2014), for instance, by establishing relationships (Mollon 2014) and trust (Groves, Bunch, and Kuehnle 2023). When patients are seen as persons their sense of safety may increase and enhance the relationship between healthcare staff and patients. These relationships can be further enhanced through person‐centred care (Péculo‐Carrasco et al. 2020) which aims to foster a sense of trust and is essential in establishing the nurse–patient relationship (Arakelian et al. 2017) According to Arakelian et al. (2017) person‐centered care during the perioperative period may reduce patients' anxiety and contribute to a faster postoperative recovery. Person‐centred care in this context depends on nurses' abilities to be both physically and emotionally present and create a sense of safety for patients. Nurse anaesthetists have several strategies for reducing patients' perceived anxiety preoperatively. These include being responsive to patients' needs, demonstrating empathy, treating patients in a friendly and respectful way, and answering any questions they may have (Clair, Engström, and Strömbäck 2020).

For patients who have undergone orthopaedic day surgery under regional anaesthesia, factors such as the opportunity for participation, a sense of control, and the relationship with the staff are important for them to feel safe (Larsson et al. 2024). Establishing the nurse–patient relationship in this specific (i.e., perioperative) setting may be demanding due to time constraints and the fact that patients are often affected by sedatives (Arakelian et al. 2017). The importance of the nurse–patient relationship may be linked to Peplau's (1991) Interpersonal Relations Theory. According to Peplau, nursing is an interpersonal process that is often therapeutic and educational. Nursing may be defined as a human relationship between a patient in need of health care and a nurse trained to address that specific need. The patient's outcome may result from that relationship (Peplau 1991). The FSS includes questions about how people perceive the staff's treatment, the information they received, and their level of trust in the staff (Rysst and Larsson 2024). These questions, along with several others in the FSS, may be directly or indirectly related to nursing and the nurse–patient relationship during the perioperative period. Thus, it may be assumed that nursing and the nurse–patient relationship may have a positive impact on patients' perceived feeling of safety and their postoperative recovery.

In summary, this study's result emphasises the importance of protecting and promoting patients' feelings of safety during the perioperative period, as it is associated with their postoperative recovery. Previous research suggests that factors such as the nurse–patient relationship and the opportunity for patients to participate in their care can promote an increased sense of safety, ultimately affecting the patient's postoperative recovery. Thus, nursing and the nurse–patient relationship are crucial in promoting patients' experiences.

### 5.1 Strengths and Limitations

This study has several limitations. The study's response rate was relatively low, 48%, which may lead to problems of generalisability. However, before the study was implemented, a power analysis was calculated to determine an appropriate sample size, thereby enhancing the ability to generalise the results and reducing the risk of selection bias (Florczak 2022). There are missing data from participants which is displayed in Tables 1 and 2. However, missing answers were not included in the regression models to avoid biased power estimates.

The distribution of gender in the current study is slightly skewed, with 61.7% women and 38.3% men, which introduces an increased risk of gender bias. The study population (*n* = 441) had a gender distribution of 56.5% women (*n* = 249) and 43.5% men (*n* = 192), which accounts for some of the observed skewness. It is a known issue that fewer men than women participate in nursing studies as well as in scientific studies in general (Galea and Tracy 2007; Polit and Beck 2013).

Due to the cross‐sectional design of the current study, the results may only suggest associations; therefore, conclusions about causality cannot be drawn. Further, since participants were invited to participate approximately 3 weeks postoperatively, there is a risk of recall bias (Althubaiti 2016). Nevertheless, since the aim was to evaluate factors influencing patients' postoperative recovery, it was considered necessary to allow a period of time to elapse after surgery to allow patients to recover to some extent. Both S‐PSR and FSS measure patients' self‐reported recovery and experiences of feeling safe, which may lead to an increased risk of social desirability bias (Althubaiti 2016). However, both instruments are evaluated regarding reliability and validity and have been proven reliable and valid (Berg et al. 2010; Rysst and Larsson 2024).

The regression models reveal relatively low levels of *r* ^2^ and adj *r* ^2^, suggesting that additional factors influence patients' perceived feeling of safety and postoperative recovery. These factors may include underlying diseases, patients' American Society of Anesthesiologists (ASA) levels, and biomedical reasons that affect recovery time from surgical procedures. Since the instruments and background questions only capture participants' self‐reported experiences, we lack information on these aspects, which is a limitation of the study. However, focusing on participants' own experiences is a strength of this study. Future research that incorporates both patients' self‐reported experiences and additional medical background factors could be valuable.

In both FSS and S‐PSR, the answer options are Likert‐scale variables. Further, the data from FSS and S‐PSR, as well as the data on levels of pain, anxiety, and nausea, are not normally distributed. However, in this study, they are treated as continuous variables in the regression models. Despite arguments against the suitability of Likert scales for such parametric analyses, compelling evidence suggests otherwise. Parametric tests, known for their robustness, have also demonstrated accurate results with non‐parametric and non‐normally distributed data. Hence, parametric tests are deemed appropriate for analysing these types of data (Norman 2010).

### 5.2 Recommendations for Future Research

This study's results show that patients undergoing orthopaedic day surgery feel safe during the perioperative period, and that feeling is associated with their postoperative recovery. To evaluate the effect of increased safety on patients' postoperative recovery, an intervention that aims to increase the perceived safety could be beneficial. As person‐centred care and the nurse–patient relationship can influence perceived safety, such an intervention could investigate the relationship between these two. The population of this study comprises patients who have undergone orthopaedic day surgery. It may be assumed that undergoing this type of surgery is not a matter of life and death, and although the recovery period may be more challenging than anticipated, it is expected to lead to a better quality of life with less pain. Therefore, it would be of value to examine the experience of another group of patients, such as people undergoing any type of cancer surgery, regarding their perception of feeling safe and the association with their postoperative recovery.

### 5.3 Implications for Policy and Practice

Strengthening the nurse–patient relationship is important, as it fosters a sense of safety during surgery. Since the feeling of safety is tied to postoperative recovery, perioperative care should prioritise creating environments and procedures where patients feel secure, addressing both physical and emotional aspects. Training nurses in effective communication, empathy, and patient engagement is key to enhancing this relationship and creating a supportive environment. Nursing curricula should emphasise the importance of strengthening patients' sense of safety, and training nurses to recognise and mitigate anxiety could enhance patient emotional safety and recovery outcomes. The importance of this is imperative to improve overall care and recovery. To identify and meet patients ‘needs, nurses should embrace the relational and emotional aspects of care, addressing both the physical and psychological needs of patients in perioperative settings. Future research should explore targeted interventions to strengthen patients’ sense of safety, while broader studies across different surgical procedures and anaesthesia methods will help establish whether these findings are applicable in other clinical contexts.

## 6 Conclusion

In this study, the perceived feeling of safety during the perioperative period could not be explained by factors such as age, gender, or level of education. In previous studies, perioperative safety has been shown to consist of attributes such as feeling involved and having a relationship with the staff. Based on the results of this study, postoperative recovery is associated with the perceived feeling of safety during the perioperative period. Anxiety in the preoperative period is associated with patients' perceived feeling of safety as well as their postoperative recovery. Thus, this study's results emphasise the importance of ensuring that people undergoing surgery feel safe, as this contributes to promoting their recovery.

## Author Contributions

All authorsd have agreed on the final version and met at least one of the following criteria recommended by the ICMJE (http://www.icmje.org/recommendations/): Made substantial contributions to study conception and design, acquired data or performed analysis, interpreted data, and drafted the article or revised it critically for important intellectual content.

## Conflicts of Interest

The authors declare no conflicts of interest.

## Statistics

The author(s) affirm that the methods used in the data analyses are suitably applied to their data within their study design and context, and the statistical findings have been implemented and interpreted correctly.

## Supporting information


Data S1.

## Data Availability

The datasets generated and analysed during the current study are available from the corresponding author upon reasonable request.
